# F-18 FDG-PET-CT in the Diagnostic of a Late Medullary Thyroid Carcinoma Recurrence in a Patient with Follicular-Papillary Thyroid Cancer

**DOI:** 10.1155/2014/741262

**Published:** 2014-02-20

**Authors:** Doina Piciu, Andra Piciu

**Affiliations:** ^1^Department of Nuclear Medicine, “Ion Chiricuţă” Institute of Oncology, 34-36 Republicii Street, 400015 Cluj-Napoca, Romania; ^2^Faculty of Medicine, “Iuliu Haţieganu” University of Medicine and Pharmacy, 6-8 V. Babes Street, 400012 Cluj-Napoca, Romania

## Abstract

Mixed medullary and follicular or papillary carcinoma of thyroid is an extremely rare tumor, characterized by coexistence of morphological and immunohistochemical features of both medullary carcinoma and follicular (or papillary) carcinoma. This case report describes for the first time in the indexed database a late recurrence of a medullary thyroid carcinoma initially diagnosed as follicular-papillary form, treated and monitored accordingly. After 14 years, a superior mediastinum tumor was discovered incidentally at a thorax computer tomography. The whole-body I-131 scan was negative and F-18 FDG-PET-CT showed glucose avidity of the tumor. The patient was operated on and the histology revealed medullary thyroid carcinoma. If there are no possibilities to have routinely extensive immunohistologic profiles, it is recommended to check the serum calcitonin, at least in any patient with confirmed thyroid carcinoma.

## 1. Introduction

Thyroid carcinoma is the most frequent endocrine cancer, accounting for about 5% of thyroid nodules [1] with a general incidence reported to be between 1.2 and 3.8 100.000 inhabitants [2, 3]. The differentiated thyroid cancers represent about 80% of all thyroid cancers [2], with a good prognosis [4, 5], while medullary, respectively, anaplastic forms, with considerable lower incidences [1, 6–9], have an aggressive evolution, frequently very severe and fast. The mixed forms are very rare and considered to be of different cell origins, with specific clinical behaviours [10–15].

Considered rare among human malignancies, the thyroid carcinoma has a very important increasing incidence in the last 10 years [3, 16], reported by studies from all over the world, with the fact that requires a new approach regarding the management of this pathology and special attention to the unusual or more aggressive forms.

## 2. Case Presentation

A 67-year-old male was admitted to our institute with the diagnostic of multiple cervical lymph nodes and right thyroid nodule. He was submitted to total thyroidectomy, with selective right lymphadenectomy for a follicular-papillary thyroid carcinoma with right cervical lymph nodes metastasis, T3N1 M0, stage III, according to the TNM of the moment [17]. At 4 weeks after the surgery, without thyroid hormone replacement the patient was evaluated prior to the metabolic irradiation with I-131. Therefore, the serum level of the thyroid-stimulating hormone (TSH) was >100 mIU/L, increased; the thyroglobulin (Tg) specific tumor marker was 13.2 ng/mL (N.V. < 0.1 ng/mL, undetectable in case of cured patients); the anti-thyroglobulin antibody (anti-Tg) was <10 IU/mL (N.V. < 34 IU/mL). There were no pathologic findings at neck ultrasound.

According to the current guidelines [1, 16], the patient was irradiated with radioiodine I-131, total activity 143 mCi (5.3 GBq). The posttherapy scan (whole-body scan - WBS I-131) done after 3 days showed minimal residual thyroid tissue in the thyroid bed.

**Figure 1:**
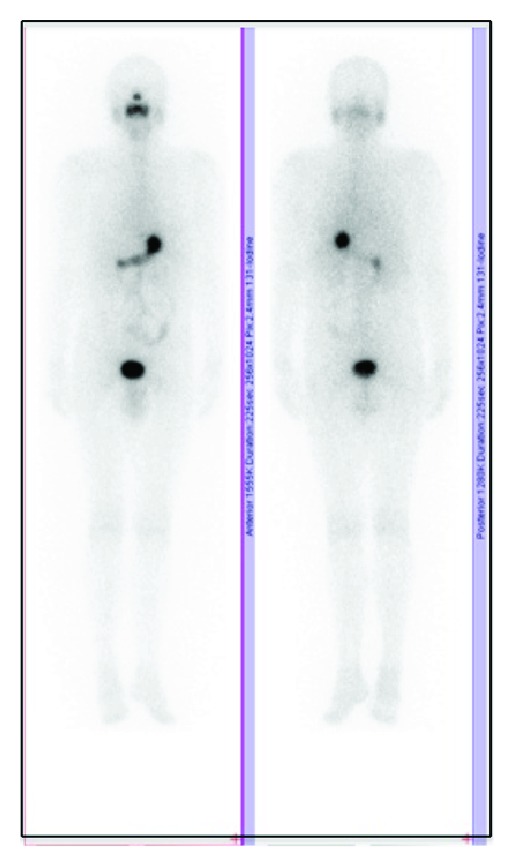
Whole-body scan with a diagnostic dose of 185 MBq I-131 showing no pathologic uptake in the body, anterior-posterior incidences.

**Figure 2:**
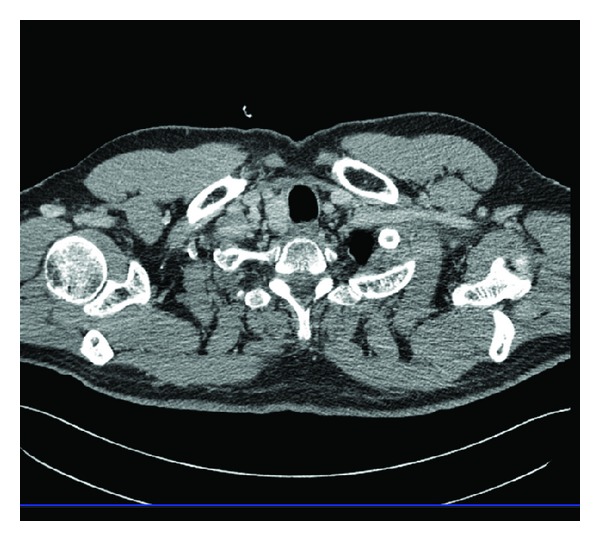
The computer tomography scan of the upper mediastinum presenting the recurrence of the tumor.

**Figure 3:**
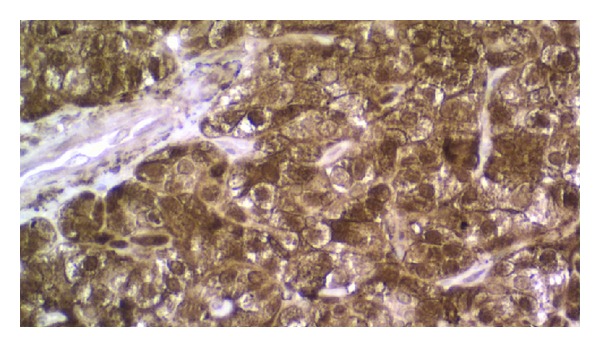
Immunohistochemical examination revealed medullary thyroid carcinoma calcitonin 200x.

The patient started the hormonal suppression with 150 *μ*g Levothyroxine/daily; the serum level of the TSH was constantly maintained at undetectable values, lower than 0.1 mIU/L (N.V. 0.4–4.2 mIU/L) and free-thyroxin (FT4) was always in normal ranges (N.V. 12–22.4 pmol/L). The patient was followed up all the time in the same department, according to the procedure applied for differentiated thyroid carcinoma: clinical exam, thyroid and neck ultrasound, and either by thyroid hormone withholding or by 2 I.M. injections of recombinant TSH (rTSH) he was checked for the specific thyroid tumor markers: TSH > 40 mIU/L, the serum value of Tg < 0.1 ng/mL (N.V. < 0.1 ng/mL), and anti-Tg < 10 kIU/L (N.V. < 141 kIU/L). The WBS I-131 under a diagnosis dose of 185 MBq of I-131 was negative (Figure 1). After the first oncological control at 6 months after therapy the patient was considered cured of thyroid cancer disease and remained under periodic observation. After 14 years he was readmitted due to a superior mediastinum tumor incidentally discovered at a thorax computer tomography (CT), made after a car accident. The CT showed in the upper mediastinum in close contact with the vessels, compressing the trachea, a tumor highly vascularized of 2.5/3 cm (Figure 2). He was checked immediately for the recurrence of DTC: neck ultrasound, Tg and anti-Tg after rhTSH stimulation, and WBS I-131 and all results were negative, excluding the relapse of the DTC. The patient was referred to F-18 FDG positron emission computer tomography PET-CT exam that showed glucose avidity of the tumor with a standard uptake value lean body mass SUV_lbm_ of 6.2 (cut-off value for malignant tissues 2.5). The patient was operated on, but, due to the invasiveness of the mass in the vessels, the surgeon removed only partially the tumor and the histology revealed medullary thyroid carcinoma (MTC) (Figure 3).

The serum calcitonin (Ct) after surgery was 872 pg/mL (N.V. < 2 pg/mL) and the carcinoembryonic antigen (CEA) 8.2 ng/mL (N.V. < 3.4 ng/mL). The EBT with a total of 60 Gy was started. At 3 months after EBT there was no clinical relapse and the neck ultrasound was negative. The head and neck, thorax, and abdominal CT were also negative, despite the abnormal calcitonin level.

**Figure 4:**
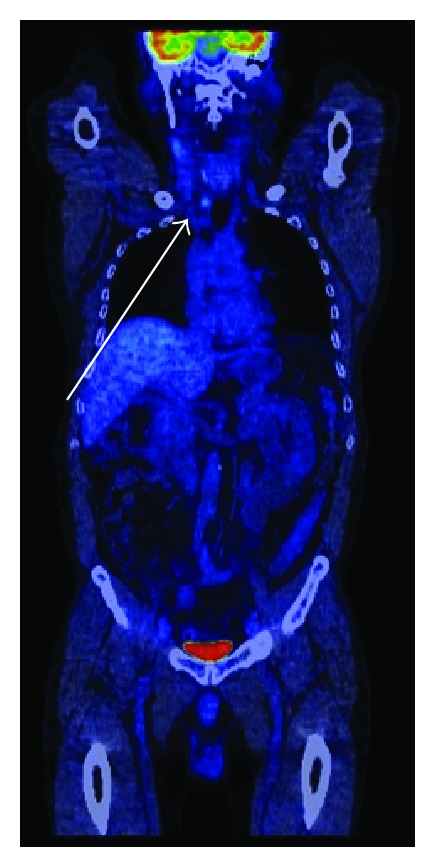
F18-FDG-PET-CT after the therapy in June 2013, showing the persistency of the tumor in the upper mediastinum with high glucose uptake and SUV_lbm_ 3.67.

It was mandatory to have an evaluation of the patient according to the pathologic level of Ct, so the F18-FDG PET-CT was requested, knowing that the previous one showed the presence of the highly metabolic tumor activity. The scan showed the persistency of the pathologic uptake in the upper mediastinum, but with a lower SUV_lbm_-3.67 and no other metastatic lesions (Figure 4). Even if in thyroid cancers, both in DTC and MTC, the F-18 FDG-PET-CT is not routinely the best option; in this case, we found a good correlation of the nuclear medicine procedure and the clinical outcome.

**Figure 5:**
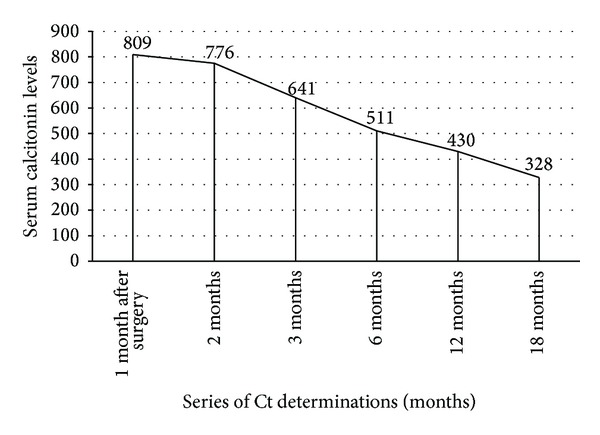
The serum levels of the calcitonin during the follow-up period January 2012–June 2013 (pg/mL).

After 2 years he is clinically disease free, with no complaints, but with high Ct level. The calcitonin values during the follow-up period January 2012–June 2013 are shown in Figure 5. The case was interpreted as a mixed form of DTC and MTC, the relapse being due to MTC form, which was misdiagnosed at the initial moment of the disease. The tumor was not associated with multiple endocrine neoplasia type 2.

We obtained the informed consent both for diagnosis and treatment and for the scientific report.

## 3. Discussion

Mixed medullary and follicular or papillary carcinoma of thyroid is an extremely rare tumor, characterized by coexistence of morphological and immunohistochemical features of both medullary carcinoma and follicular (or papillary) carcinoma. While specific identification of mixed forms by FNA may be difficult, it should be emphasized that adequate sampling in conjunction with the proper immunostaining panel could have highlighted the different aspects of the mixed tumor. Different molecular mechanisms for mixed thyroid tumors have been suggested, like the possible association with the uncommon polymorphism G691S of the RET protooncogene, but the origins of this rare tumor entity remains unclear [18].

Some authors published results, which provide strong evidence that the follicular and medullary components in mixed forms are not derived from a single progenitor cell. They demonstrated that follicular structures in these forms are often oligo/polyclonal and more frequently exhibit hyperplastic than neoplastic histological features, indicating that at least a subset of the tumors are composed of a medullary thyroid carcinoma containing hyperplastic follicles [10]. We should note also the possibility of an existing specific immunohistochemical profile negative for thyroglobulin and positive for calcitonin. A few patients with this variant have been reported in the literature, mainly diagnosed by immunohistochemical features of the tumor and these cases should be distinguished from the mixed medullary-follicular thyroid carcinomas and medullary carcinomas with entrapped follicles [12]. There are also tumors which are considered medullary carcinomas with thyroglobulin immunoreactivity [19], since they do not fulfill the WHO criteria for “mixed medullary-follicular carcinomas,” it is very difficult to set accurate results.

The case reported in this paper is a particular situation, for the first time described in the indexed database, due to the late relapse and to the different forms of cancer, which was diagnosed during the recurrence. It is completely improbable that the initial DTC has been transformed in a MTC form, the real situation being a misdiagnosis at the first presentation. The lack of immune profile on the initial histological specimens, more than 14 years ago, is the most important cause of the false consideration of the case as pure DTC. Also, the absence of routine determination of serum calcitonin, especially after the result of histology showing the follicular variant of the papillary thyroid carcinoma, led to the misinterpretation of the case.

There are some important comments related to this case.

1. Is it necessarily to have routinely an extensive immunohistochemical profile on histological specimens? If yes, the specialists must provide the most suitable panel able to cover even these particular situations.
2. Should we always do calcitonin in any patient with thyroid carcinoma? Maybe yes, considering the aggressiveness of the MTC and the cost-effectiveness analysis of this context.
3. Should we perform CT on a routine basis in DTC? It is well known that DTC is monitored at best standards by ultrasound, WBS I-131, and specific tumor markers; CT, MRI, and PET-CT do not bring more and better information in this pathology. We should not forget also the high curability of the DTC and the long-life follow-up of these patients, so a 6–10 mSv effective dose/examination at CT must be carefully managed, according to side effects of the ionizing radiations.
4. The serum calcitonin is abnormal, but in dynamic decreasing: what is the most suitable therapeutically decision? Chemotherapy, clinical trials (new drugs), or follow-up “wait and see.” Considering that the patient has no complaints, we decided the conservative attitude and after 2 years of monitoring the Ct is constantly decreasing and the disease is stable. In this case, the results of F18-FDG-PET-CT showing a SUV_lbm_ not significantly increased had a crucial importance in the management of the disease.

## 4. Conclusion

If there are no possibilities to have routinely extensive immunohistologic exams at patients with thyroid carcinoma, it is recommended to check the serum calcitonin and the association of different histological profiles which are misdiagnosed, being able to lead to severe outcomes.

## References

[1] Pacini F, Schlumberger M, Dralle H, Elisei R, Smit JW, Wiersinga W (2006). European consensus for the management of patients with differentiated thyroid carcinoma of the follicular epithelium. *European Journal of Endocrinology*.

[2] Elisei R, Molinaro E, Agate L (2010). Are the clinical and pathological features of differentiated thyroid carcinoma really changed over the last 35 years? Study on 4187 patients from a single Italian institution to answer this question. *The Journal of Clinical Endocrinology and Metabolism*.

[3] Ferlay J, Steliarova-Foucher E, Lortet-Tieulent J (2013). Cancer incidence and mortality patterns in Europe: estimates for 40 countries in 2012. *European Journal of Cancer*.

[4] Haddad RI (2013). New developments in thyroid cancer. *Journal of the National Comprehensive Cancer Network*.

[5] Tuttle RM, Tala H, Shah J (2010). Estimating risk of recurrence in differentiated thyroid cancer after total thyroidectomy and radioactive iodine remnant ablation: using response to therapy variables to modify the initial risk estimates predicted by the new American Thyroid Association staging system. *Thyroid*.

[6] McLeod DS, Sawka AM, Cooper DS (2013). Controversies in primary treatment of low-risk papillary thyroid cancer. *The Lancet*.

[7] Muntean V, Domsa I, Zolog A (2013). Incidental papillary thyroid microcarcinoma: is completion surgery required?. *Chirurgia*.

[8] Pedrazzini L, Baroli A, Marzoli L, Guglielmi R, Papini E (2013). Cancer recurrence in papillary thyroid microcarcinoma: a multivariate analysis on 231 patients with a 12-year follow-up. *Minerva Endocrinologica*.

[9] Piciu D, Piciu A, Irimie A (2012). Papillary thyroid microcarcinoma and ectopic papillary thyroid carcinoma in mediastinum: a case report. *Clinical Nuclear Medicine*.

[10] Volante M, Papotti M, Roth J (1999). Mixed medullary-follicular thyroid carcinoma: molecular evidence for a dual origin of tumor components. *American Journal of Pathology*.

[11] Ben Salah R, Mekni A, Doghri R (2012). A mixed medullary-follicular thyroid carcinoma discovered by fine needle aspiration. *La Tunisie Medicale*.

[12] Çakir M, Altunbaş H, Balci MK, Karayalçin Ü, Karpuzoğlu G (2002). Medullary thyroid carcinoma, follicular variant. *Endocrine Pathology*.

[13] Zoroquiain P, Torres J, Goni I, Fernandez L, Solar A (2012). True mixed medullary papillary carcinoma of the thyroid: a case report with low blood calcitonin levels. *Endocrine Pathology*.

[14] Hanna AN, Michael CW, Jing X (2011). Mixed medullary-follicular carcinoma of the thyroid: diagnostic dilemmas in fine-needle aspiration cytology. *Diagnostic Cytopathology*.

[15] Luboshitzky R, Dharan M (2004). Mixed follicular-medullary thyroid carcinoma: a case report. *Diagnostic Cytopathology*.

[16] Cooper DS, Doherty GM, Haugen BR (2009). Revised American thyroid association management guidelines for patients with thyroid nodules and differentiated thyroid cancer. *Thyroid*.

[17] Shaha AR (2007). TNM classification of thyroid carcinoma. *World Journal of Surgery*.

[18] Maruna P, Duskova J, Limanova Z, Dvoraková S, Vaclavikova E, Bendlova B (2008). Mixed medullary and follicular cell carcinoma of the thyroid in a 71-year-old man with history of malignant melanoma. *Medical Science Monitor*.

[19] Kos M, Separović V, Sarcević B (1995). Medullary carcinoma of the thyroid: histomorphological, histochemical and immunohistochemical analysis of twenty cases. *Acta Medica Croatica*.

